# Supplementary material for: Pan-cancer analysis of mRNA stability for decoding tumour post-transcriptional programs
Source: Commun Biol. 2022 Aug 20;5:851. doi: 10.1038/s42003-022-03796-w (PMC9392771; doi:10.1038/s42003-022-03796-w)
Supplement: Supplementary file 2 — Supplementary Information [file 42003_2022_3796_MOESM2_ESM.pdf]

Supplementary Figures for

Pan-cancer analysis of mRNA stability for decoding tumour post-transcriptional programs

Gabrielle Perron[1,2], Pouria Jandaghi[2], Elham Moslemi[2], Tamiko Nishimura[2], Maryam Rajaee[2], Rached Alkallas[1,2], Tianyuan Lu[1,2], Yasser Riazalhosseini[1,2], Hamed S. Najafabadi[1,2,*]

1 Department of Human Genetics, McGill University, Montreal, QC H3A 1B1, Canada
2 McGill Genome Centre, Montreal, QC H3A 0G1, Canada

* Corresponding author: H. S. Najafabadi, hamed.najafabadi@mcgill.ca

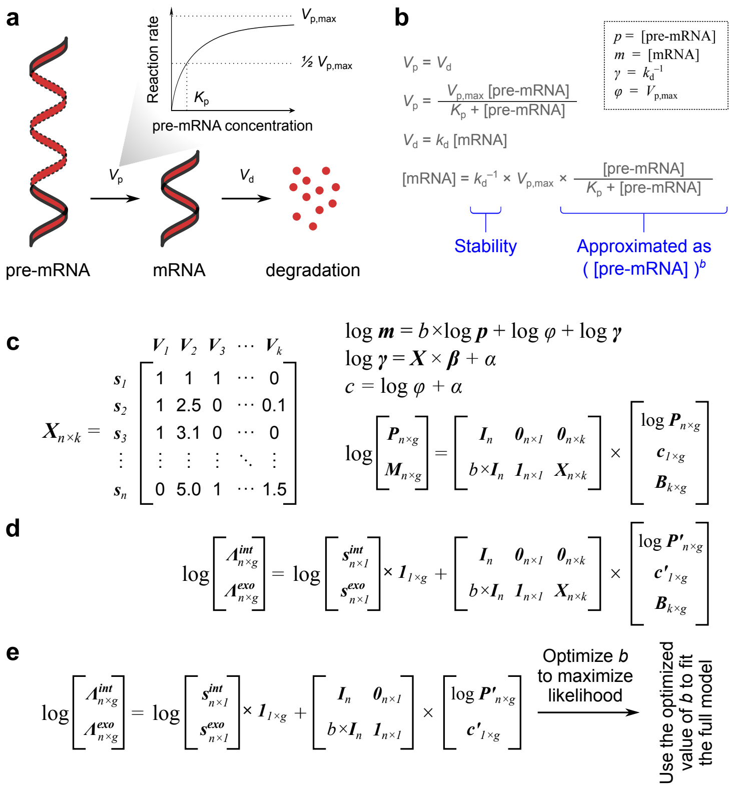

Supplementary Figure 1. The DiffRAC model. (**a**) Schematic presentation of mRNA processing and decay steps based on the model proposed by Alkallas et al. [1]. (**b**) This model leads to an equation that can be approximated as a power-law relationship between stability and the abundance of pre-mRNA and mature mRNAs. Panels **a**-**b** are modified from Alkallas et al. [1] (reproduced under CC-BY license, https://creativecommons.org/licenses/by/4.0/). The notations that are used in this paper to present different quantities in this kinetic model are shown on the top-right. (**c**) DiffRAC aims to model mRNA stability (γ) as a function of a given a design matrix X, which specifies sample characteristics (left). To do this, we start from the power-law relationship between pre-mRNA and mature mRNA abundance and convert it to log-scale (top-right). Logarithm of stability is then modeled as a linear function of the design variables X with coefficients β. This system of equations can be expressed using matrix operations, as shown on the bottom-right. X: the $n \times k$ design matrix for k variables across n samples. P: the $n \times g$ matrix of pre-mRNA abundance for g genes across n samples. M: the $n \times g$ matrix of mature mRNA abundance. I_n: the $n \times n$ identity matrix. B: the $k \times g$ matrix of coefficients representing the effect of each of the k variables on the stability of each of the g genes. The parameter b is the bias-term (same as in panel **b**), which is assumed to be shared across genes and samples. The parameter c is a gene-specific factor that represents the combined effect of maximum processing rate (φ) and baseline RNA stability (α, i.e. the intercept for the stability function). (**d**) DiffRAC estimates the latent variables in this model by fitting them to the observed intronic (*int*) and exonic (*exo*) read counts. For this purpose, the logarithm of the mean (λ) of read counts is modeled as a function of pre-mRNA and mature mRNA abundances (from panel **c**), in addition to sample-specific library size factors (s) for intronic and exonic counts. Λ: the $n \times g$ matrix of the mean of intronic (*int*) or exonic (*exo*) read counts, for g genes across n sampels. Note that here P is replaced with P', as the fitted values will represent the combination of P and a latent, gene-specific scaling factor for intronic reads. Similarly, c is replaced with c' since the fitted values will represent the combination of c and a latent, gene-specific scaling factor for exonic reads. (**e**) To estimate b, DiffRAC first fits a model that does not include the effect of stability, and optimizes b to maximize the joint likelihood of intronic and exonic read counts. In other words, b is chosen to maximize the likelihood of data in the absence of changes in mRNA stability. This optimized b is then used to fit the full model in order to examine whether addition of mRNA stability terms to the model significantly improves the fit. DiffRAC uses DESeq2 [2] for library size estimation, model fitting, and likelihood calculation. See **Methods** for more details.

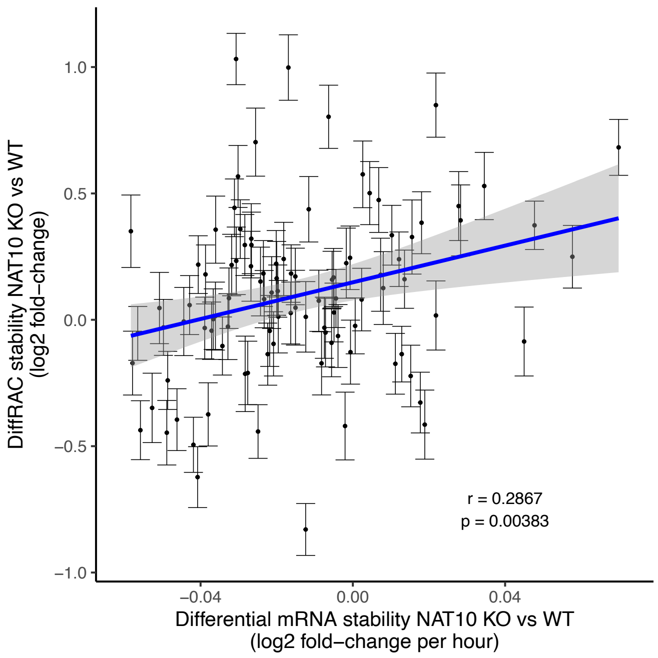

Supplementary Figure 2. Additional DiffRAC benchmarking in NAT10 knockout cells. Comparison of DiffRAC estimates of differential stability, using RNA-seq data from a NAT10 knockout in HeLa cells compared to WT (data from ref. [4]), vs. ground-truth stability as calculated using BRIC-seq data (see **Methods** for more details). Genes with low read counts (< 5) were first filtered out, and the 100 genes with the smallest sum of DiffRAC SEMs and ground-truth estimates SEMs were kept for calculation of Pearson correlation. Error bars represent DiffRAC SEMs. The Pearson correlation coefficient and p-value are shown on the plot.

a $\log \boldsymbol{m} = b \times \log \boldsymbol{p} + \log \varphi + \log \boldsymbol{\gamma}$

$\log \boldsymbol{\gamma} = \boldsymbol{X} \times \boldsymbol{\beta} + \alpha \qquad c = \log \varphi + \alpha$

$\log \boldsymbol{p} = \boldsymbol{X} \times \boldsymbol{\omega} + \rho$

$$\log \begin{bmatrix} \boldsymbol{P}_{n\times g} \\ \boldsymbol{M}_{n,g} \end{bmatrix} = \begin{bmatrix} \boldsymbol{1}_{n\times 1} & \boldsymbol{X}_{n\times k} & \boldsymbol{0}_{n\times 1} & \boldsymbol{0}_{n\times k} \\ \boldsymbol{1}_{n\times 1} & b\times\boldsymbol{X}_{n\times k} & \boldsymbol{1}_{n\times 1} & \boldsymbol{X}_{n\times k} \end{bmatrix} \times \begin{bmatrix} \boldsymbol{\rho}_{1\times g} \\ \boldsymbol{\Omega}_{k\times g} \\ \boldsymbol{c}_{1\times g} \\ \boldsymbol{B}_{k\times g} \end{bmatrix}$$

b

$$\log \begin{bmatrix} \Lambda^{int}_{n\times g} \\ \Lambda^{exo}_{n\times g} \end{bmatrix} = \log \begin{bmatrix} s^{int}_{n\times 1} \\ s^{exo}_{n\times 1} \end{bmatrix} \times \boldsymbol{1}_{1\times g} + \begin{bmatrix} \boldsymbol{1}_{n\times 1} & \boldsymbol{X}_{n\times k} & \boldsymbol{0}_{n\times 1} & \boldsymbol{0}_{n\times k} \\ \boldsymbol{1}_{n\times 1} & b\times\boldsymbol{X}_{n\times k} & \boldsymbol{1}_{n\times 1} & \boldsymbol{X}_{n\times k} \end{bmatrix} \times \begin{bmatrix} \boldsymbol{\rho'}_{1\times g} \\ \boldsymbol{\Omega}_{k\times g} \\ \boldsymbol{c'}_{1\times g} \\ \boldsymbol{B}_{k\times g} \end{bmatrix}$$

c

$$\log \begin{bmatrix} \Lambda^{int}_{n\times g} \\ \Lambda^{exo}_{n\times g} \end{bmatrix} = \log \begin{bmatrix} s^{int}_{n\times 1} \\ s^{exo}_{n\times 1} \end{bmatrix} \times \boldsymbol{1}_{1\times g} + \begin{bmatrix} \boldsymbol{1}_{n\times 1} & \boldsymbol{X}_{n\times k} & \boldsymbol{0}_{n\times 1} \\ \boldsymbol{1}_{n\times 1} & b\times\boldsymbol{X}_{n\times k} & \boldsymbol{1}_{n\times 1} \end{bmatrix} \times \begin{bmatrix} \boldsymbol{\rho'}_{1\times g} \\ \boldsymbol{\Omega}_{k\times g} \\ \boldsymbol{c'}_{1\times g} \end{bmatrix}$$

Optimize *b* to maximize likelihood → Use the optimized value of *b* to fit the full model

d

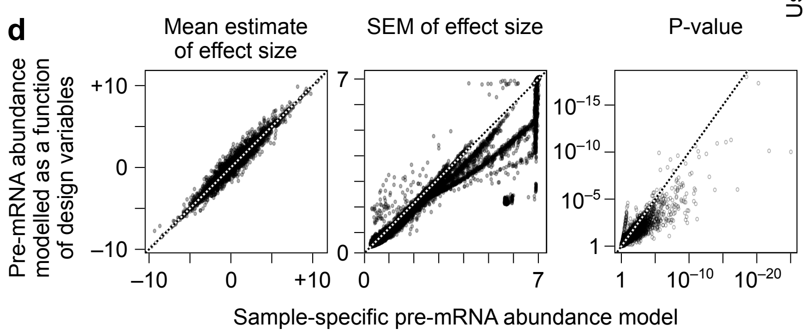

Supplementary Figure 3. A simplified version of the DiffRAC model. (**a**) In this simplified model, instead of assuming sample-specific latent pre-mRNA abundances, the pre-mRNA abundance is also modeled as a linear function of the design matrix *X*, with the coefficients *ω* and intercept (baseline pre-mRNA abundance) equal to *ρ*. *Ω*: the *k*×*g* matrix of coefficients representing the effect of each of the *k* variables on the pre-mRNA abundance of each of the *g* genes. Other variables are the same as **Supplementary Figure 1**. (**b**) Similar to **Supplementary Figure 1D**, DiffRAC fits this model to the observed intronic and exonic read counts. Note that *ρ* and *c* are replaced with *ρ'* and *c'* since the fitted values will also contain latent gene-specific scaling factors for intronic and exonic counts, respectively. (**c**) Similar to **Supplementary Figure 1E**, the bias parameter *b* is estimated by maximizing the likelihood for a model that assumes constant mRNA stability for each gene across samples. This optimized *b* is then used in the full model. (**d**) DiffRAC produces comparable statistics when sample-specific latent variables are included for pre-mRNA abundance (i.e. the full model; x-axis) or pre-mRNA abundance is modeled as a function of design variables (i.e. the simplified model; y-axis). Data correspond to DiffRAC analysis of differential stability between mouse ES cells and ES cells differentiated to terminal neurons [3].

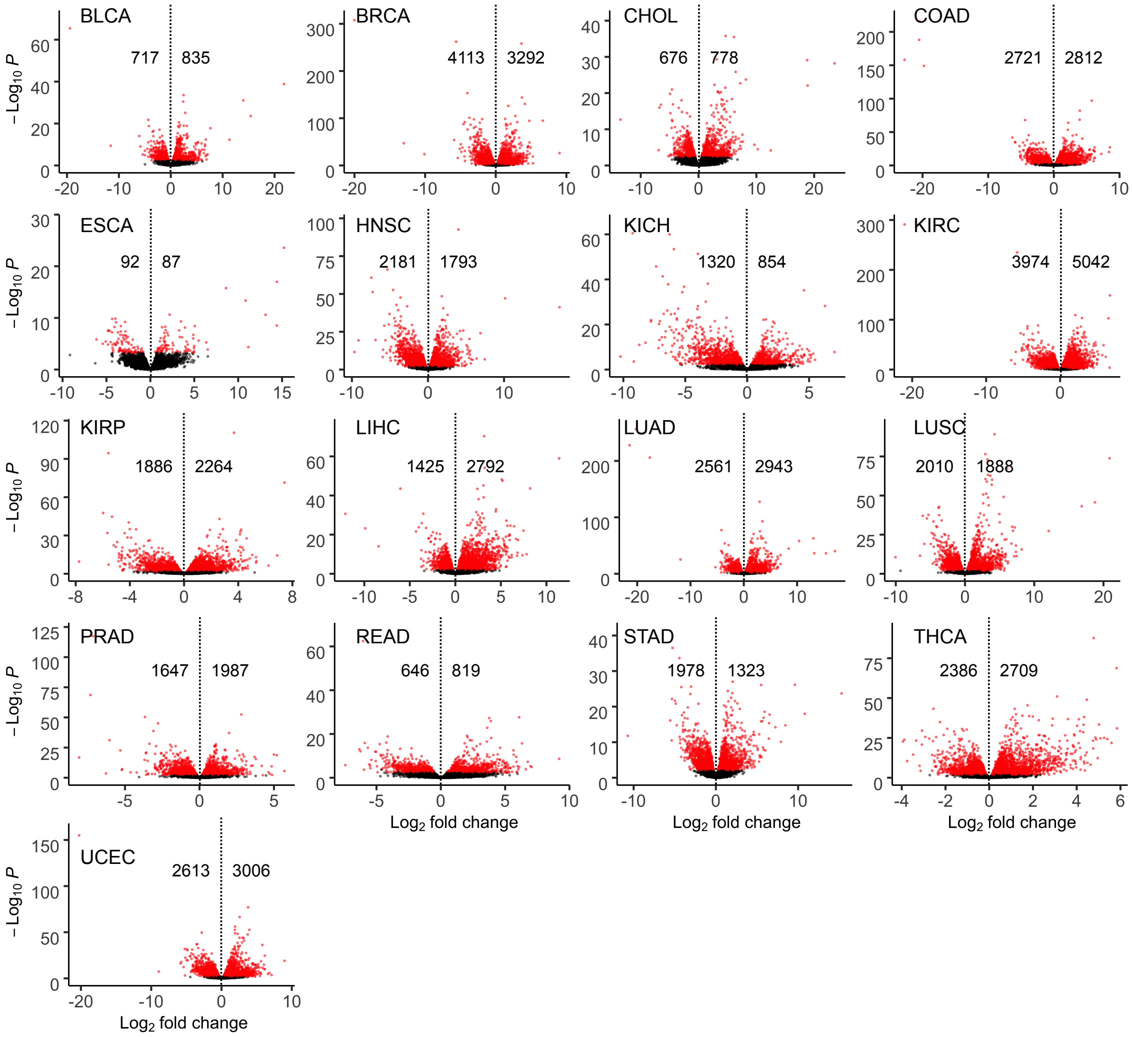

Supplementary Figure 4. Differential mRNA stability in TCGA cancers. (**a**) Volcano plots showing differential stability, between tumour and normal samples, in each of the 18 TCGA cancers analyzed by DiffRAC. The x-axis represents the log2 fold-change of mRNA stability (T/N) and the y-axis represents the log10 P. Genes that pass FDR < 0.05 are colored in red. The number of significantly stabilized (right) and destabilized (left) genes are indicated on the plots.

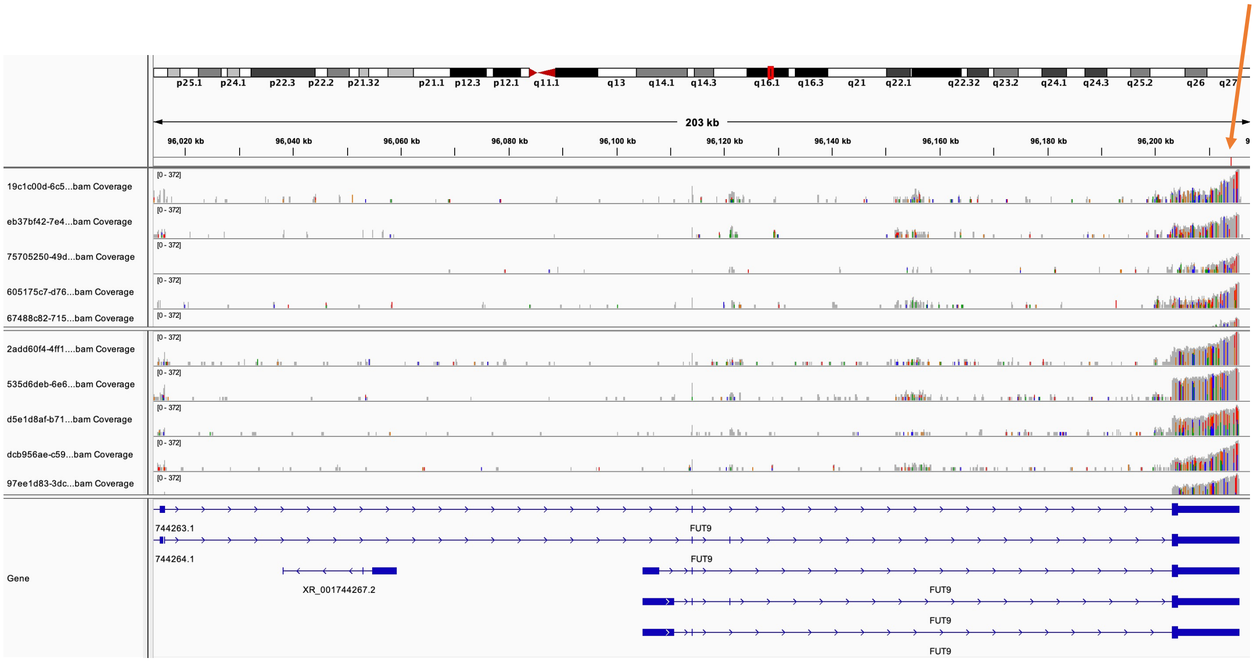

Supplementary Figure 5. Representative changes in exonic and intronic coverage for a differentially stabilized gene in cancer. Integrative Genomics Viewer (IGV) visualization of BAM files for the sequencing data of 5 TCGA-GBM tumor samples and 5 normal samples, focusing on the *FUT9* gene. Read coverage is shown in the logarithmic scale. The RBFOX1 binding site is shown by the arrow.

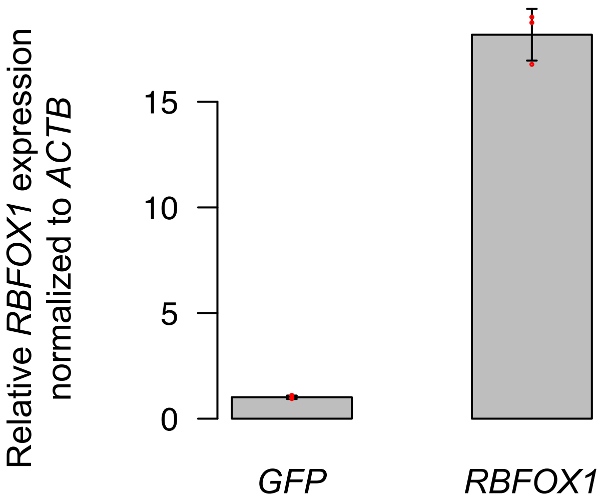

Supplementary Figure 6. qRT-PCR confirmation of RBFOX1 overexpression in the human glioblastoma cell line A172. (**a**) Relative *RBFOX1* expression normalized to *ACTB* in stable cell lines overexpressing GFP (left) or *RBFOX1* (right) (see **Methods** for more details). Average $2^{-\Delta\Delta Ct}$ values (n=3) are shown. Error bars correspond to the standard deviation and individual data points are shown in red.

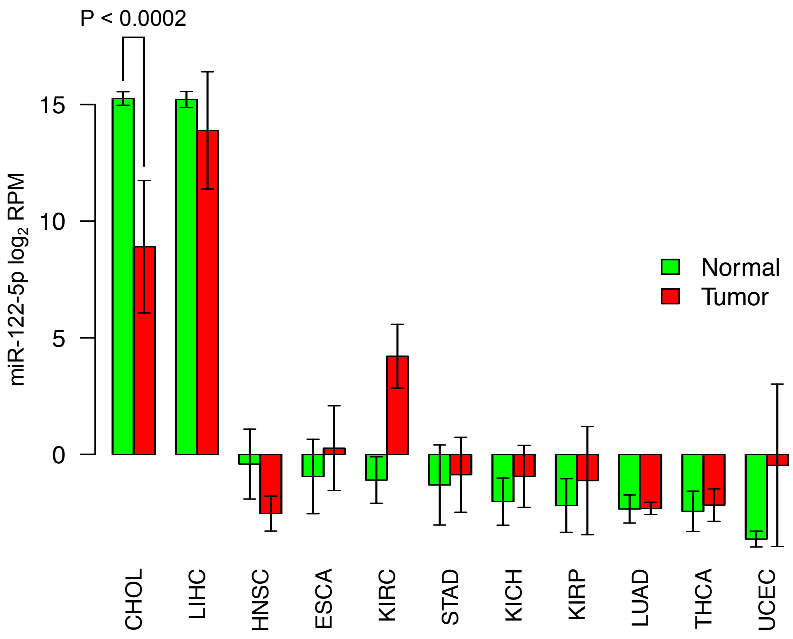

Supplementary Figure 7. Expression of miR-122 across cancer types. Only TCGA cancer types with miR-122 measurement in patient-matched normal and tumour tissues are included. P-value is based on paired t-test. Error bars represent the standard deviation.

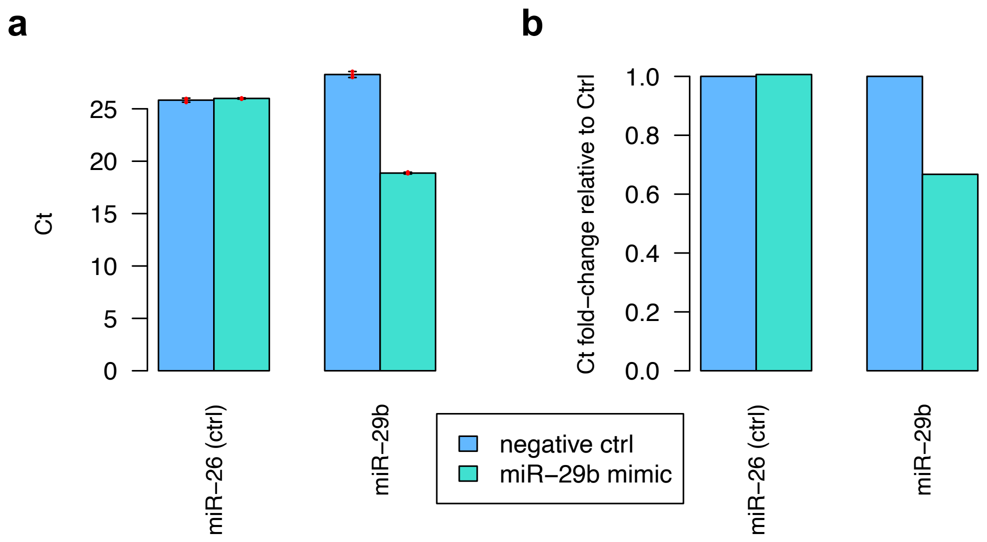

Supplementary Figure 8. qRT-PCR confirmation of miR-29-mimic expression in 786-O cells. (**a**) Average Ct values (n=3) are shown. Error bars in black correspond to the standard deviation. Individual data points are shown in red. Negative control corresponds to transfection with a control mimic. Right: change in miR-29b expression after transfection with miR-29b-mimic. Left: change in miR-26 expression (as an internal control). Note that smaller C_t values correspond to higher expression of the assayed miRNA. (**b**) Similar to (a), but showing relative expression (fold-change relative to control) instead of raw Ct values.

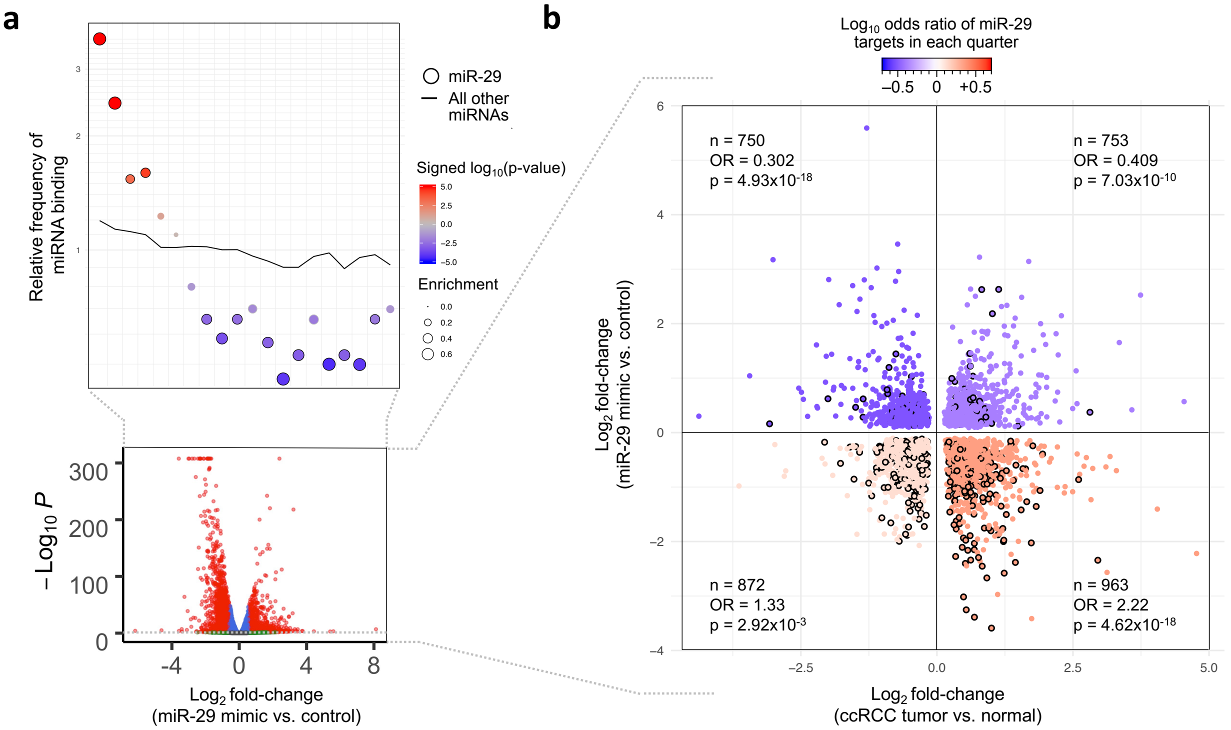

Supplementary Figure 9. MiR-29 regulon dysregulation after miR-29 overexpression in A-498 cells and its comparison to TCGA-KIRC cohort. (**a**) Enrichment of miR-29 targets among genes that are downregulated after transfection of a miR-29 mimic in A-498 cells. See **Figure 6C** for plot details. (**b**) Similar to **Figure 6D**, this figure shows the enrichment of miR-29 binding sites, relative to other miRNA binding sites, in genes that are stabilized in TCGA-KIRC tumours (relative to normal tissue) and downregulated after miR-29 mimic expression in A-498 cells (n = 2) relative to controls (n = 2). See **Figure 6D** for plot details.

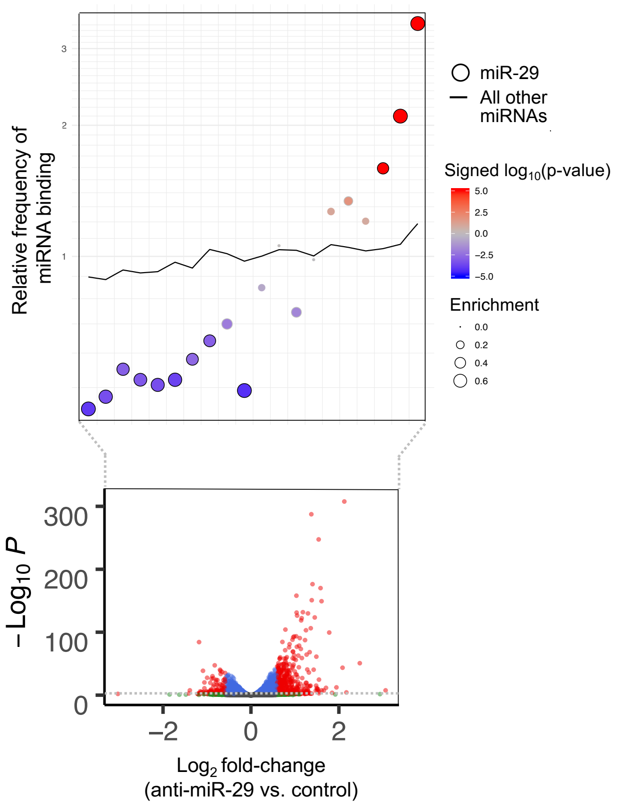

Supplementary Figure 10. miR-29 regulon dysregulation upon miR-29 inhibition. Enrichment of miR-29 targets among genes that are upregulated after transfection of a miR-29 inhibitor in ACHN cells (similar to **Figure 6C** and S9A). See **Figure 6C** for plot details.

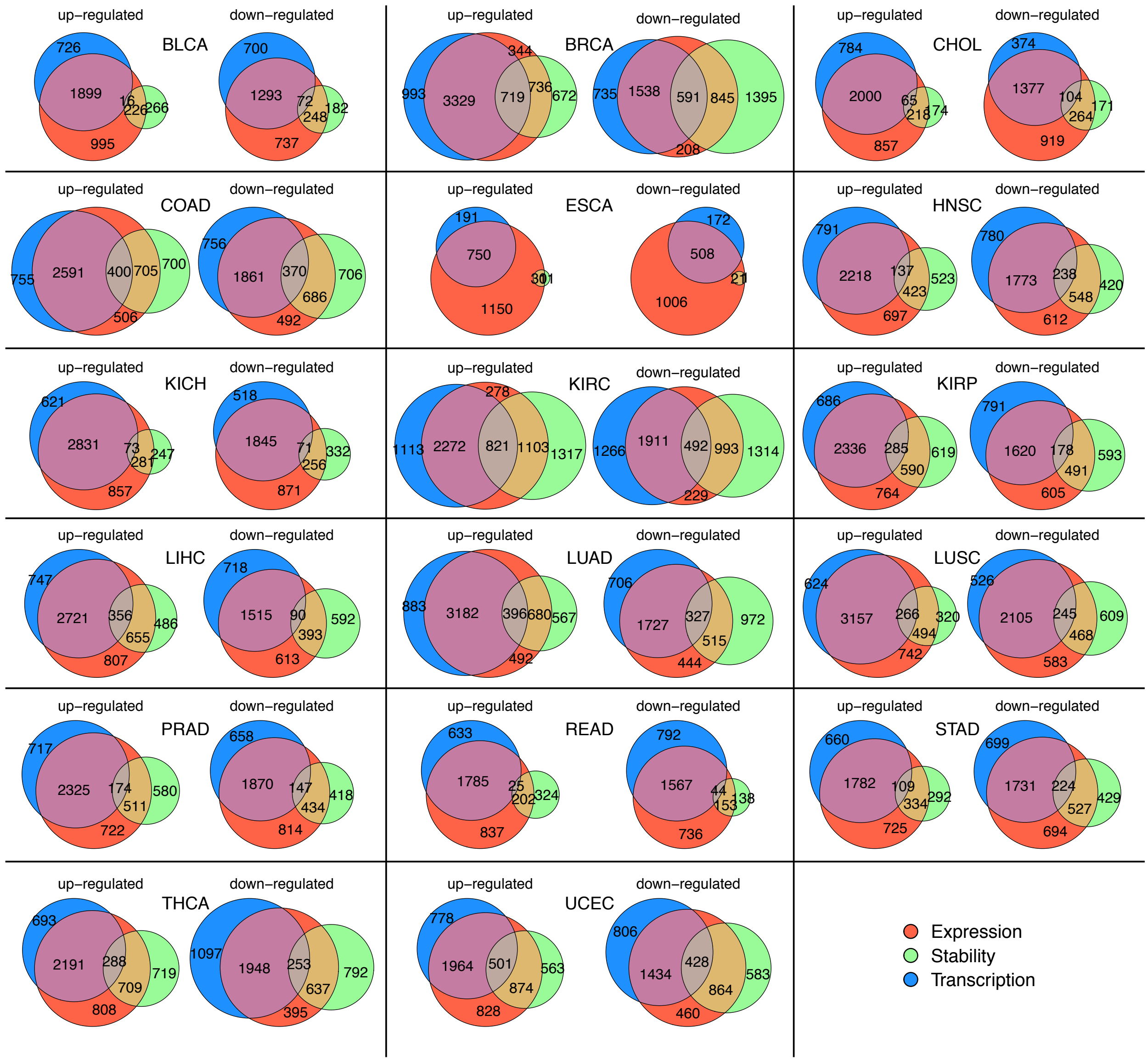

Supplementary Figure 11. Overlap of differentially expressed genes with genes that show differential stability or differential transcription in each cancer. For each cancer type, differentially up-regulated or down-regulated genes are plotted separately. In each case, differential expression was assayed using DESeq2 based on read counts downloaded from TCGA. Differential stability is based on DiffRAC. Differential transcription is based on DiffRAC variables that correspond to pre-mRNA abundance. In all cases, significant hits are identified based on FDR < 0.05.

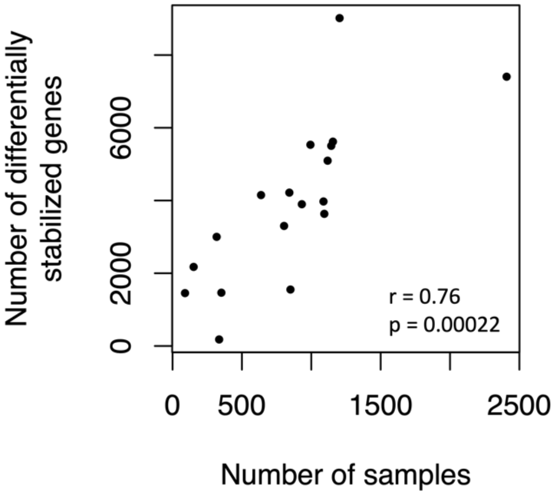

Supplementary Figure 12. The number of significantly differentially stabilized genes in TCGA datasets is positively correlated with the sample size. The Pearson correlation coefficient and p-value are shown on the plot.

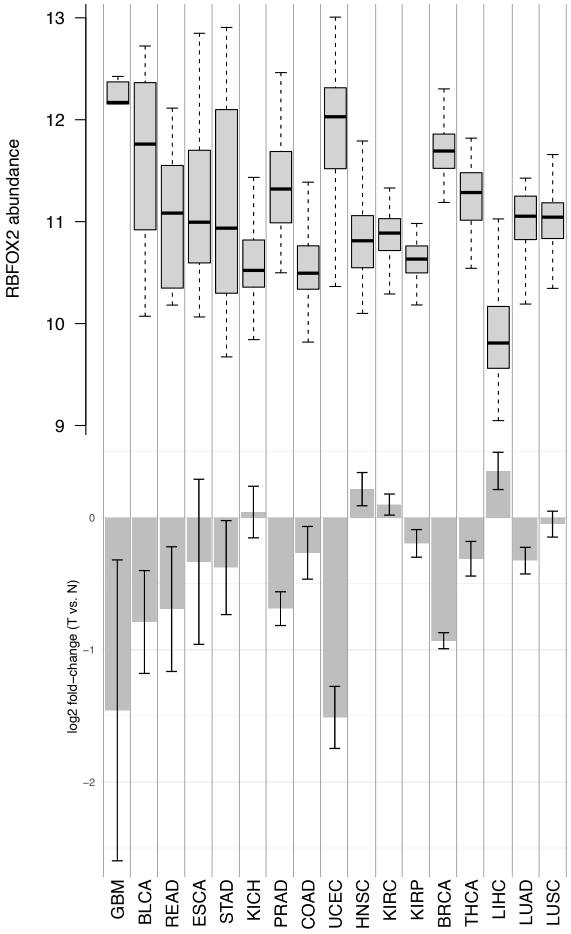

Supplementary Figure 13. ***RBFOX2*** **expression across TCGA cancer types.** Similar to **Figure 5A-B.** (**a**) The box plot (top) shows the *RBFOX2* log2(RSEM) gene expression, retrieved from Firebrowse (http://firebrowse.org/), in normal tissue samples. The bar plot (bottom) illustrates the average log fold-change of *RBFOX2* expression in tumors compared to normal samples (error bars represent SEM).

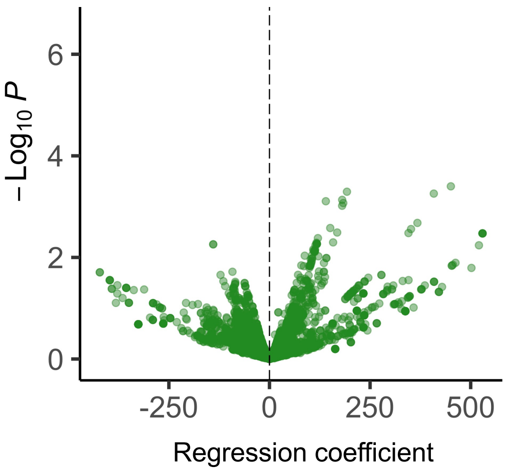

Supplementary Figure 14. Association between disruption of mRNA stability and loss of heterozygosity (LOH) of RNA decay genes. Volcano plot showing the coefficients from a linear regression testing the association between the degree of disruption of mRNA stability (defined as the number of significantly differentially stabilized genes in each tumor) and LOH of RNA decay genes. We also corrected for the confounding effect of patient age, sex, total number of LOH events in the samples, and tumor purity. Cell cycle genes were excluded from the analysis **(see Methods)**. The x-axis represents the regression coefficients for the LOH variable and the y-axis represents the log10P. Genes that are coloured in green did not pass FDR < 0.05. We defined RNA decay genes using a manually curated list of 969 genes involved in mRNA degradation, including genes in the KEGG pathway "RNA degradation" [5], in addition to 52 Gene Ontology (GO) biological processes [6, 7] related to mRNA degradation and miRNA pathways. For each RNA decay gene and each cancer type, we tested the association between LOH of that gene and the degree of disruption of mRNA stability in that cancer type (3077 pairs of RNA decay genes and cancer types, with at least 20 mutated samples, were considered). LOH calls were derived from ABSOLUTE copy number data obtained from [8].

Supplementary References

1. Alkallas R, Fish L, Goodarzi H, Najafabadi HS: **Inference of RNA decay rate from transcriptional profiling highlights the regulatory programs of Alzheimer's disease.** *Nat Commun* 2017, **8:**909.
2. Love MI, Huber W, Anders S: **Moderated estimation of fold change and dispersion for RNA-seq data with DESeq2.** *Genome Biol* 2014, **15:**550.
3. Gaidatzis D, Burger L, Florescu M, Stadler MB: **Analysis of intronic and exonic reads in RNA-seq data characterizes transcriptional and post-transcriptional regulation.** *Nat Biotechnol* 2015, **33:**722-729.
4. Arango D, Sturgill D, Alhusaini N, Dillman AA, Sweet TJ, Hanson G, Hosogane M, Sinclair WR, Nanan KK, Mandler MD, et al: **Acetylation of Cytidine in mRNA Promotes Translation Efficiency.** *Cell* 2018, **175:**1872-1886 e1824.
5. Kanehisa M, Goto S: **KEGG: kyoto encyclopedia of genes and genomes.** *Nucleic Acids Res* 2000, **28:**27-30.
6. Ashburner M, Ball CA, Blake JA, Botstein D, Butler H, Cherry JM, Davis AP, Dolinski K, Dwight SS, Eppig JT, et al: **Gene ontology: tool for the unification of biology. The Gene Ontology Consortium.** *Nat Genet* 2000, **25:**25-29.
7. Gene Ontology C: **The Gene Ontology resource: enriching a GOld mine.** *Nucleic Acids Res* 2021, **49:**D325-D334.
8. Taylor AM, Shih J, Ha G, Gao GF, Zhang X, Berger AC, Schumacher SE, Wang C, Hu H, Liu J, et al: **Genomic and Functional Approaches to Understanding Cancer Aneuploidy.** *Cancer Cell* 2018, **33:**676-689 e673.
