# Supplementary material for: Pan-cancer analysis of mRNA stability for decoding tumour post-transcriptional programs
Source: Commun Biol. 2022 Aug 20;5:851. doi: 10.1038/s42003-022-03796-w (PMC9392771; doi:10.1038/s42003-022-03796-w)
Supplement: Supplementary file 3 — Description of Additional Supplementary Files [file 42003_2022_3796_MOESM3_ESM.pdf]

Description of Additional Supplementary Files

File name: Supplementary Data 1
Description: (**A**) Estimation of differential mRNA stability using the RNA-seq data from mouse embryonic stem cells and terminal neurons, along with ground-truth transcript half-life measurements after transcriptional blockage with actinomycin D. DiffRAC estimates are listed in columns 2-7. Ground-truth half-life measurements are in column 8. (**B**) Estimation of differential mRNA stability using RNA-seq data from a NAT10 knockout in HeLa cells compared to WT, along with ground-truth stability as calculated using BRIC-seq data (see **Methods** for more details). Genes with low read counts (< 5) are filtered out. DiffRAC estimates are listed in columns 2-7. Ground-truth stability data and associated SEMs are in columns 8-9.

File name: Supplementary Data 2
Description: Pan-cancer analysis of differential mRNA stability across TCGA cancers, comparing tumours to normal tissue. DiffRAC estimates for each cancer type are provided in separate sheets.

File name: Supplementary Data 3
Description: **A**) Differential stability comparing the MDA-MB-231 and MDA-LM2 cell lines. (**B**) Differential stability comparing highly metastatic relative to poorly metastatic PDX models of breast cancer.

File name: Supplementary Data 4
Description: Clinical data and tumour purity for TCGA samples that were used for analysis of stage- and/or grade-associated stability changes.

File name: Supplementary Data 5
Description: (**A**) Genes with significant mRNA stability changes associated with tumour stage in at least one of the 11 TCGA cancers for which this type of classification was available. Note that no significant associations were identified for READ, and therefore this cancer type has no entries in this table. (**B**) Similar to **A**, for cancerous/pre-cancerous cells (see **Methods** for more details).

File name: Supplementary Data 6
Description: Genes with significant mRNA stability changes associated with tumour grade in at least one of the four cancer types for which this type of classification was available.

File name: Supplementary Data 7
Description: (**A**) Differential gene expression in RBFOX1 knockdown in terminally differentiated neurons. DESeq2 estimates are provided. (**B**) Differential gene expression following RBFOX1 overexpression in the human glioblastoma cell line A172. DESeq2 estimates are provided.

File name: Supplementary Data 8
Description: (**A**) Differential gene expression in the miR-29 mimic relative to control mimic expression in 786-O cells. DESeq2 estimates are provided. (**B**) Similar to **A**, for miR-29 mimic relative to control mimic expression in A-498 cells. (**C**) Similar to **A**, for miR-29 inhibition in ACHN cells (relative to control).
