# Supplementary material for: Pan-cancer analysis of mRNA stability for decoding tumour post-transcriptional programs
Source: Commun Biol. 2022 Aug 20;5:851. doi: 10.1038/s42003-022-03796-w (PMC9392771; doi:10.1038/s42003-022-03796-w)
Supplement: Supplementary file 12 — Reporting Summary [file 42003_2022_3796_MOESM12_ESM.pdf]

nature portfolio

Corresponding author(s): Hamed S. Najafabadi

Last updated by author(s): Jul 19, 2022

Reporting Summary

Nature Portfolio wishes to improve the reproducibility of the work that we publish. This form provides structure for consistency and transparency in reporting. For further information on Nature Portfolio policies, see our Editorial Policies and the Editorial Policy Checklist.

Statistics

For all statistical analyses, confirm that the following items are present in the figure legend, table legend, main text, or Methods section.

n/a	Confirmed	
☐	☒	The exact sample size (*n*) for each experimental group/condition, given as a discrete number and unit of measurement
☐	☒	A statement on whether measurements were taken from distinct samples or whether the same sample was measured repeatedly
☐	☒	The statistical test(s) used AND whether they are one- or two-sided<br>*Only common tests should be described solely by name; describe more complex techniques in the Methods section.*
☐	☒	A description of all covariates tested
☐	☒	A description of any assumptions or corrections, such as tests of normality and adjustment for multiple comparisons
☐	☒	A full description of the statistical parameters including central tendency (e.g. means) or other basic estimates (e.g. regression coefficient) AND variation (e.g. standard deviation) or associated estimates of uncertainty (e.g. confidence intervals)
☐	☒	For null hypothesis testing, the test statistic (e.g. F, t, r) with confidence intervals, effect sizes, degrees of freedom and P value noted<br>*Give P values as exact values whenever suitable.*
☒	☐	For Bayesian analysis, information on the choice of priors and Markov chain Monte Carlo settings
☒	☐	For hierarchical and complex designs, identification of the appropriate level for tests and full reporting of outcomes
☐	☒	Estimates of effect sizes (e.g. Cohen's *d*, Pearson's *r*), indicating how they were calculated

Our web collection on statistics for biologists contains articles on many of the points above.

Software and code

Policy information about availability of computer code

Data collection	No software was used
Data analysis	DiffRAC (custom): https://github.com/csglab/DiffRAC; HISAT2 (v2.2.1); HTSeq (v0.11.1); samtools (v1.12); R; DESeq2 (v1.34.0)

For manuscripts utilizing custom algorithms or software that are central to the research but not yet described in published literature, software must be made available to editors and reviewers. We strongly encourage code deposition in a community repository (e.g. GitHub). See the Nature Portfolio guidelines for submitting code & software for further information.

Data

Policy information about availability of data

All manuscripts must include a data availability statement. This statement should provide the following information, where applicable:

- Accession codes, unique identifiers, or web links for publicly available datasets
- A description of any restrictions on data availability
- For clinical datasets or third party data, please ensure that the statement adheres to our policy

Data generated during this study are included in this published article and its supplementary files. Additional data and analysis files are available at http://csg.lab.mcgill.ca/sup/pancancer_stability/ and/or via Zenodo (doi:10.5281/zenodo.4404547). RNA-seq data from the miR-29 mimic and inhibitor expression experiments are available via GEO under accession GSE145088. RNA-seq data from the RBFOX1 overexpression experiment are also available via GEO under

accession GSE201639. The results published here are in part based on data generated by the TCGA Research Network: https://www.cancer.gov/tcga. Other data used in this paper are available via their source publications as indicated in the article.

Human research participants

Policy information about studies involving human research participants and Sex and Gender in Research.

Reporting on sex and gender	N/A
Population characteristics	N/A
Recruitment	N/A
Ethics oversight	N/A

Note that full information on the approval of the study protocol must also be provided in the manuscript.

Field-specific reporting

Please select the one below that is the best fit for your research. If you are not sure, read the appropriate sections before making your selection.

☒ Life sciences ☐ Behavioural & social sciences ☐ Ecological, evolutionary & environmental sciences

For a reference copy of the document with all sections, see nature.com/documents/nr-reporting-summary-flat.pdf

Life sciences study design

All studies must disclose on these points even when the disclosure is negative.

Sample size	Sample size for TCGA cohort analysis depended on publicly available data. No statistical analysis was performed to select the sample sizes for RNA-seq experiments (biological replicates were used).
Data exclusions	Two RNA-seq samples related to the miR-29 mimic experiment performed in A-498 cells were excluded due to potential sample mislabeling.
Replication	To ensure reproducibility for RNA-seq experiments, biological replicates were used and/or the findings were replicated in other cell lines.
Randomization	N/A
Blinding	N/A

Reporting for specific materials, systems and methods

We require information from authors about some types of materials, experimental systems and methods used in many studies. Here, indicate whether each material, system or method listed is relevant to your study. If you are not sure if a list item applies to your research, read the appropriate section before selecting a response.

Materials & experimental systems

n/a	Involved in the study	
☒	☐	Antibodies
☐	☒	Eukaryotic cell lines
☒	☐	Palaeontology and archaeology
☒	☐	Animals and other organisms
☒	☐	Clinical data
☒	☐	Dual use research of concern

Methods

n/a	Involved in the study	
☒	☐	ChIP-seq
☒	☐	Flow cytometry
☒	☐	MRI-based neuroimaging

Eukaryotic cell lines

Policy information about cell lines and Sex and Gender in Research

Cell line source(s)	Cell lines were obtained from American Type Culture Collection (ATCC; Rockville, MD, USA)

March 2021

Authentication	No independent authentication (cell lines were purchased from ATCC)
Mycoplasma contamination	All cell lines tested negative for mycoplasma contamination.
Commonly misidentified lines (See ICLAC register)	N/A
